# Effect of Two Remineralizing Agents on Dentin Microhardness of Non-Caries Lesions

**DOI:** 10.30476/dentjods.2023.95663.1883

**Published:** 2023-12-01

**Authors:** Haleh Heshmat, Haleh Kazemi, Maryam Hoorizad Ganjkar, Farhad Chaboki, Mahoor Shokri, Mohamad Javad Kharazifard

**Affiliations:** 1 Dept. of Restorative Dentistry, Member of Dental Material Research Center, Islamic Azad University of Medical Sciences, Tehran, Iran; 2 Dept. of Restorative Dentistry, School of Dentistry, Islamic Azad University of Medical Sciences, Tehran, Iran; 3 Postgraduate Student, School of Dentistry, Islamic Azad University of Medical Sciences, Tehran, Iran; 4 School of Dentistry, Islamic Azad University of Medical Sciences, Tehran, Iran; 5 Dental Research Center, Tehran University of Medical Sciences, Tehran, Iran

**Keywords:** CPP-ACP, Dentin, Hardness, Hydroxyapatites, Tooth Remineralization

## Abstract

**Statement of the Problem:**

The prevalence of non-carious dentin lesions is on the rise mainly due to improved life expectancy. Successful management of these lesions is often challenging, and given that dentin can be remineralized, adverse consequences due to progression of these lesions can be prevented or minimized as such.

**Purpose:**

This study aimed to assess the effect of casein phosphopeptide amorphous calcium phosphate (CPP-ACP) and Remin-Pro remineralizing agents on dentin microhardness of non-carious dentin lesions.

**Materials and Method:**

This *in vitro*, experimental study evaluated 36 extracted sound human premolars. The teeth were decoronated at the cementoenamel junction. Enamel was removed, and dentin was exposed at the cervical third of the buccal surface. The primary microhardness of dentin was then measured. The teeth, standardized in terms of dentin microhardness, then underwent demineralization by acid etching and were subjected to microhardness test again. They were then randomized into three groups for treatment with CPP-ACP, Remin-Pro, and artificial saliva (control), and dentin microhardness was measured for the third time after treatment. Data were analyzed using ANOVA.

**Results:**

Within group comparisons showed a significant difference in microhardness at the three time points in all three groups (*p*< 0.005). Between-group comparisons revealed that the microhardness of the three groups was not significantly different at baseline or after demineralization. However, the microhardness of the three groups was significantly different after the intervention (*p*= 0.000). Pairwise comparisons revealed significantly higher microhardness in the CPP-ACP group than the other two groups (*p*= 0.003). Remin-Pro and the control groups were not significantly different in this respect (*p*= 0.340).

**Conclusion:**

CPP-ACP can be used for remineralization of non-caries dentin lesions; however, Remin-Pro does not appear to be effective for this purpose.

## Introduction

The prevalence of non-carious dentin lesions is on the rise mainly due to improved life expectancy. These lesions have been reported in 56% of males over 75 years of age [ 1 ]. Also, they have a higher prevalence in patients with a history of head and neck radiotherapy [ 1 ]. These lesions can adversely affect the long-term survival of the teeth [ 1 ]. Non-caries dentin lesions are multi-factorial, and can lead to tooth hypersensitivity, plaque accumulation, and caries development. If not treated, they can compromise the structural integrity of the teeth and can even affect the pulp vitality. Successful management of these lesions is often challenging, and given that dentin can be remineralized in these lesions, adverse consequences due to progression of these lesions can be prevented or minimized as such [ 2 ]. 

Due to the significance of conservative dentistry, researchers have long been in search of enamel and dentin remineralizing agents [ 3 - 5 ]. Remineralization of tooth structure often occurs as the result of an increase in the levels of calcium, HPO4, and fluoride ions as well as the buffering agents in the saliva [ 6 - 7 ]. 

Casein phosphopeptide amorphous calcium phosphate (CPP-ACP) is derived from the milk protein and is used as a remineralizing agent. The optimal efficacy of CPP-ACP for remineralization of enamel lesions has been well documented [ 8 ]. CPP bonds to enamel and releases calcium and phosphate whenever required. The released calcium and phosphate ions penetrate into the enamel crystals and increase the density and hardness of hydroxyapatite crystals [ 9 - 10 ]. 

Remin-Pro is another remineralizing agent, which unlike CPP-ACP, contains calcium and phosphate in the form of hydroxyapatite. It also contains fluoride and Xylitol. Remin-Pro Forte is a newer generation of Remin-Pro, which also contains ginger and turmeric extracts. It not only remineralizes the tooth structure, but also affects the soft tissue. It has been confirmed that Remin-Pro can positively enhance enamel remineralization [ 11 - 13 ]. However, studies regarding its efficacy for dentin remineralization are limited [ 14 ]. Both CPP-ACP and Remin-Pro remineralize the enamel structure by deposition of calcium and phosphate ions, and reinforcing the structure of hydroxyapatite crystals. Thus, they may be able to remineralize the dentin as well, due to the presence of hydroxyapatite in dentin structure [ 15 ]. 

The microhardness of enamel and dentin indicates the mineral content of their surface. Several studies have measured microhardness as an indicator for the degree of mineralization of enamel and dentin [ 11 - 12 , 14 ]. Thus, this study aimed to assess whether it is effective to use CPP-ACP and Remin-Pro remineralizing agents on dentin microhardness in non-caries dentin lesions or not.

## Materials and Method

### Samples collection

This *in vitro* experimental study evaluated 36 human premolar teeth extracted as part of orthodontic treatment or due to poor periodontal prognosis. The teeth were sound and had no carious lesion, restoration, crack, wear, or hypoplasia. The study protocol was approved by the Ethics Committee of our university (IR.IAU. DENTAL.REC.1398.001). The sample size was calculated to be 12 in each group according to a study by Liang *et al* [ 16 ], assuming alpha=0.05, beta=0.2, standard deviation of the mean microhardness to be 65, and effect size of 0.57 using one-way ANOVA power analysis feature of PASS 11 software. 

### Samples preparation

The teeth had been extracted within the past 1 month, and had been stored in 0.1% thymol solution at room temperature. Tissue residues were removed by a scalpel, and the teeth were cleaned with a prophy brush and low-speed hand-piece under running water. The teeth were then decoronated by a diamond disc and stored in distilled water. Next, enamel of the cervical third of the buccal surface of the teeth was removed by a diamond bur to expose dentin. For the purpose of standardization of specimens, the exposed dentin was polished with 600 -grit abrasive discs (Sof-Lex; 3M ESPE, St. Paul, MN, USA). The specimens were then mounted in auto-poly-merizing acrylic resin in the form of blocks measuring 2×3cm such that the dentin surface remained exposed.

### Vickers microhardness test

The baseline dentin microhardness of each mounted specimen was then measured by a digital Vickers microhardness tester (Bareiss, USA), and all specimens were standardized regarding baseline microhardness as much as possible. The microhardness was measured at 3 points, and the mean value was calculated and reported as the mean Vickers hardness number. 

### Demineralization procedure of samples

Next, the specimens were randomized into three groups (n=12) of CPP-ACP (GC Tooth Mousse; GC America Inc.; USA), Remin-Pro (Voco GmbH, Germany), and artificial saliva (control) by block randomization. To induce demineralization, the specimens were etched with 37% phosphoric acid (Morva-Etch, Iran) for 15 s [ 16 ], rinsed with saline for 1 min, and stored in a buffering solution with a pH of 7.4 at room temperature [ 14 ]. All specimens underwent microhardness test again after the etching process.

### Intervention

Next, the specimens were treated with CPP-ACP and Remin-Pro according to the manufacturers’ instructions for 5 min daily, for a total of 15 days [ 17 - 18 ]. The control specimens were stored in artificial saliva at 37°C for 15 days after the second microhardness test. It should be noted that all specimens in the CPP-ACP and Remin-Pro groups were stored in artificial saliva at the time intervals between the interventions. The composition of the artificial saliva was water, glycine, sodium hyaluronate, propylene glycol, lysine, and proline. The artificial saliva was refreshed daily. The final microhardness of the specimens was measured in all three groups after 15 days using the same Vickers microhardness tester as explained earlier.

### Statistical analysis

Repeated measures ANOVA was used to compare the microhardness at different time points within the three groups. One-way ANOVA was applied for pairwise comparisons. All statistical analyses were performed using SPSS version 25 at 0.05 level of significance. 

## Results

Table 1 presents the measures of central dispersion for the microhardness (Vickers hardness number) of the three groups. Repeated measures ANOVA showed a significant difference in microhardness at different time points within each of the three groups (*p*= 0.001).

**Table 1:** Measures of central dispersion for the microhardness (Vickers hardness number) of the three groups. CPP-ACP: Casein phosphopeptide amorphous calcium phosphate

Group	Time point	Maximum	Minimum	Meanstd. deviation
Group 1 (Control)	Baseline	40.60	12.00	25.005.9
	After acid etching	24.80	12.30	16.893.7
	Final	16.40	6.90	11.702.65
Group 2 (CPP-ACP)	Baseline	40.60	14.80	27.736.32
	After acid etching	40.60	10.20	16.486.62
	After intervention	22.20	4.00	14.174.30
Group 3 (Remin-Pro)	Baseline	40.10	14.20	31.059.96
	After acid etching	25.80	8.10	15.573.72
	After intervention	15.10	5.70	10.662.03
*p* Value	0.000			

Between-group comparison of microhardness revealed a significant difference in microhardness of the three groups as well (*p*< 0.05). Thus, microhardness of the three groups was compared with each other separately at each time point using ANOVA. The results showed no significant difference in microhardness among the three groups at baseline (*p*> 0.05), or after acid etching and demineralization (*p*= 0.482). 

However, the difference in microhardness of the three groups was significant in the final measurement after the intervention (*p*= 0.000). Thus, one-way ANOVA was applied for pairwise comparisons of microhardness of the groups at each time point. The results showed significant differences in microhardness between the control (group 1) and the CPP-ACP (group 2) (*p*= 0.003) and Remin-Pro (group 3) (*p*= 0.000) such that the group 2 had significantly higher microhardness than the other two groups. 

However, the difference in this respect was not significant between the group 3 and control (*p*= 0.340). Neither the CPP-ACP nor the Remin-Pro could return the baseline microhardness of the specimens.

## Discussion

This study assessed the effect of CPP-ACP and Remin-Pro remineralizing agents on dentin micro-hardness of non-caries lesions. Remin-Pro and the control groups were not significantly different in this regard. Hydroxyapatite is the main constituent of Remin-Pro. The GC Tooth Mousse contains nano-scale CPP-ACP, which may explain simpler deposition of ions in-between the collagen fibers in this group while the hydroxyapatite crystals in the composition of Remin-Pro cannot remineralize the dentin as well as they remineralize the enamel. Since the dentin structure is different from the enamel structure in terms of presence of dentinal tubules and collagen fibers, it appears that some modifications are required to be made in the structure of hydroxyapatite crystals in the composition of Remin-Pro in order to be able to induce dentin remineralization.

The mineral content of enamel and dentin determines their microhardness [ 19 ]. Thus, microhardness test was performed in this study to assess the remineralization of dentin. The microhardness test is widely used for assessment of enamel remineralization. For instance, Denelon *et al*. [ 20 ] used the microhardness test to assess enamel remineralization by CPP-ACP, and Kamath *et al*. [ 18 ] used the microhardness test to assess the remineralizing capacity of Remin-Pro. Despite the availability of studies on the efficacy of CPP-ACP and Remin-Pro for enamel remineralization, studies on their efficacy for dentin remineralization are limited. Poggio *et al*. [ 21 ] evaluated the remineralization of enamel and dentin with CPP-ACP using atomic force microscopy and scanning electron microscopy. They reported that CPP-ACP had a significant protective effect against enamel and dentin demineralization; however, this effect was greater for the enamel. Studies regarding the effects of CPP-ACP and Remin-Pro on the enamel are controversial. Some authors have reported higher efficacy of CPP-ACP [ 11 ] while others have shown the equal efficacy of the two for enamel remineralization [ 12 , 22 ]. 

In the present study, 37% phosphoric acid was used for demineralization since it affects the hydroxyapatite and weakens the mineral structure of dentin, simulating demineralization in the clinical setting [ 14 , 16 ]. Remineralization occurs as the result of penetration of calcium and phosphate ions in-between the crystals, causing their recrystallization. Naturally, these ions are provided by the saliva [ 23 ]. Fluoride is the most widely known agent to enhance the remineralization process and prevent enamel demineralization [ 24 ]. At present, the efficacy of dairy products for enhancement of remineralization and prevention of demineralization has been the topic of many investigations. Both GC Tooth Mousse and Remin-Pro are recommended for patients with tooth hypersensitivity and high risk of enamel erosion in order to enhance enamel remineralization [ 12 , 17 ]. 

Greater efficacy of CPP-ACP for dentin remineralization in the present study may be due to the more controlled release of calcium and phosphate ions from the CPP-ACP paste compared with Remin-Pro. In other words, CPP-ACP bonds to areas in higher need of calcium and phosphate ions, and gradually releases these ions over time [ 9 - 10 ]. It should be noted that despite the higher efficacy of CPP-ACP for dentin remineralization compared with the other two groups, all groups experienced a reduction in microhardness during the experiment, which has not been reported by studies conducted on the enamel [ 12 , 20 , 24 ]. This finding may be attributed to several reasons such as lower percentage of minerals in dentin, and the differences in the structure of enamel and dentin, causing enhanced demineralization of dentin over time. This is also the case in dentin caries since carious lesions have higher speed of progression in dentin, especially in root dentin.

It should be noted that this study had an *in vitro* design. Thus, generalization of results to the clinical setting must be done with caution.

## Conclusion

Within the study limitations, the results showed that the CPP-ACP (in GC Tooth Mousse) can reinforce the remineralization of demineralized dentin while Remin-Pro did not show similar effect. In the future, this study can be performed on patients with non-caries dentin lesions (like erosion) as an in vivo study.
